# 

**Published:** 1872-10

**Authors:** 


### CONTENTS:

ORIGINAL COMMUNICATIONS:

ARTICLE I. Retroversion of the Unimpregnated Womb. By A. Reeves Jackson, M.D., Chicago .......... 577

ARTICLE II. Electro-Therapeutics. By A. P. Peck, M.D., Chicago .... 588

ARTICLE III. Monstrosity. By J. W. Brooks, M.D., Chicago .......... 593

ARTICLE IV. A Case of Poisoned Wound. By G. C. Paoli, M.D., Chicago .......... 595

ARTICLE V. Iritis—Atropia—Paracentesis—Topical Use of Calomel—Aneurism of the Orbit—Sympathetic Ophthalmitis. By E. L. Holmes, M.D., Chicago .......... 596

SELECTIONS:

Cholera .......... 601

Address on English Recollections of a German Surgeon .......... 605

Statistical Details of Four Years Experience in respect to the Form of Amaurosis supposed to be due to Tobacco .......... 618

EDITORS' BOOK TABLE .......... 633

EDITORIAL .......... 635

LOOT .......... 639

